# New guidance for self-harm: an opportunity not to be missed

**DOI:** 10.1192/bjp.2023.113

**Published:** 2023-11

**Authors:** Faraz Mughal, Fiona M Burton, Harriet Fletcher, Karen Lascelles, Rory C O’Connor, Sarah Rae, Alex Thomson, Nav Kapur

**Affiliations:** MPhil, FRCGP, GP and national institute for health and care research doctoral fellow, School of Medicine, Keele University, Keele, ST5 5BG, UK; practising NHS GP and NIHR Doctoral Fellow who researches the assessment, treatment, and prevention of mental health conditions, self-harm, and suicide in primary care; MRCS(Glas), FRCEM, Emergency Medicine & Major Trauma Consultant, Queen Elizabeth University Hospital, Greater Glasgow & Clyde; consultant in Emergency Medicine and Major Trauma based in the Queen Elizabeth University Hospital Glasgow; MRCPsych, Consultant Psychiatrist in Psychotherapy, Leeds and York Partnership NHS Foundation Trust; Consultant Psychiatrist in Psychotherapy at Leeds and York Partnership NHS Foundation Trust; RMN, MSc, Nurse Consultant, Oxford Health NHS Foundation Trust; mental health nurse consultant specialising in the areas of self-harm and suicide; Professor of Health Psychology, Suicidal Behaviour Research Laboratory, School of Health and Wellbeing, University of Glasgow, Glasgow, UK; Professor of Health Psychology, President of International Association for Suicide Prevention and he leads the Suicidal Behaviour Research Laboratory at Glasgow University; Independent expert by experience; experience of co-occurring mental health conditions and self-harming behaviours. She draws on this lived experience to improve care through her involvement in research; FRCPsych, Consultant Liaison Psychiatrist, Northwick Park Hospital, Central and North West London NHS Foundation Trust; Consultant Liaison Psychiatrist at Northwick Park Hospital, Central and North West London NHS Foundation Trust. He is Interim Chair of the RCPsych Liaison Psychiatry Faculty; MD, FRCPsych, Professor of Psychiatry and Population Health, Centre for Mental Health and Safety, Manchester Academic Health Sciences Centre, University of Manchester; Mersey Care NHS Foundation Trust; NIHR Greater Manchester Patient Safety Research Collaboration, University of Manchester, Manchester, UK; Professor of Psychiatry and Population Health/Honorary Consultant at University of Manchester and Mersey Care NHS Foundation Trust, undertaking self-harm and suicide research for 25 years

Self-harm is a common problem in patients who seek help from clinical services and is associated with a heightened risk of suicide.(1) In recent years, there have been increases in self-harm particularly in young people, but also in other groups such as middle-aged men.(2) Reducing rates remains a key national suicide prevention priority.(2) Although there has been some progress in the quality of mental health care people receive for self-harm in the past few decades, there is still considerable room for improvement.

The National Institute for Health and Care Excellence (NICE) has updated its guidance for self-harm with the publication of the new 2022 ‘Self-harm: assessment, management, and preventing recurrence’ [NG225] guideline.(3) The new guidance incorporates the latest evidence and combines the previous self-harm guidelines (short and long-term management of self-harm-CG16 and CG133) so that the recommendations are available in a single publication for the first time. The guideline also considers the role of the wider system (primary care, emergency care, education, and criminal justice settings) because people who harm themselves can present to a range of settings.

In this editorial we, as clinical, academic, and lived experience members of the 2022 NICE guideline committee, highlight and discuss what, in our view, are the key guideline recommendations (generated through evidence synthesis and consensus) for mental health professionals when caring for people after self-harm. We also consider some of the implementation challenges. Our aim is not to summarise the entire guidance, and we acknowledge our approach may have led to some bias in the recommendations included here. The full guideline is readily accessible via the NICE website.

## The need for empathy

Mental health professionals work across a diverse range of service settings and will need to approach and interpret the guidance in different ways, but much of the guideline is concerned with fundamental principles of care. A consistent theme throughout is the need to provide compassionate, non-judgemental, and empathic care to people who have self-harmed. That is not to say all care for self-harm is currently lacking in these elements. There are examples of excellent care but equally examples of care which is suboptimal. These overarching principles are perhaps even more important to implement for people who frequently self-harm. They describe negative experiences of receiving mental health treatment which can have a harmful impact on help seeking and risk of suicide.(4) All staff should have access to appropriate supervision, and mental health services need to ensure that the training for practitioners is developed with and co-delivered by people with lived experience to emphasise sensitive person centred care. Family involvement is key and people who have self-harmed and their family members or carers should receive information about self-harm, care and treatment options, support agencies and resources, as well as self-care, tailored to individual needs.

## No place for aversive treatments

It is regrettable that past approaches to treatment of self-harm have included shaming, punitive treatment, or withholding care based on misguided theories related to eliciting attention. Although these approaches have no place in 21st Century practice, contemporary lived experience accounts demonstrate that they still occur. The guideline restates the principle that there is no place for aversive or punitive treatment, excluding patients, or withholding the care that they need, and this extends to the use of criminal punishment as a deterrent.(3)

## Psychosocial assessment not risk assessment

The guideline states that all people who have self-harmed should receive a psychosocial assessment at the earliest opportunity from a trained mental health professional to identify the circumstances of the episode and strengths and needs of the person. This assessment should be conducted with hope and optimism, and should not be unduly delayed for medical treatment, with liaison psychiatry teams pivotal in delivering this in emergency departments within general hospitals. Some patients have described not being offered an assessment, which is alarming. The guideline includes detail of what the assessment should cover but perhaps even more important than the ‘what’ (the content of the assessment) is the ‘how’: it should be carried out using a compassionate and collaborative approach to develop a shared understanding of why self-harm has occurred.

Mental health professionals are urged to not use risk assessment tools and scales, and to not stratify risk into low, medium, or high to predict future self-harm repetition or suicide. The guidance also clearly states that risk tools or risk stratification should not determine who should or should not be discharged or offered treatment. The positive predictive values of these tools and categories are poor: they provide false clinical reassurance and lead people in low and medium risk groups being excluded from care.(3) Instead, age-appropriate mental health professionals should focus on the person’s individual circumstances, strengths, and needs and develop a risk formulation. This drawing together of the clinical narrative will directly inform care and treatment rather than assessment being an end in itself. Although it is potentially a huge implementation challenge clinical leaders could help to facilitate a move from a ‘risk-focused’ to a ‘safety-focused’ culture in self-harm services by recognising the therapeutic benefit of sensitive comprehensive assessments rather than approaches which reduce individual experience to a risk category; moving from tick box approaches to holistic risk formulation; and addressing patients’ needs rather than treating their risk scores.

## Aftercare and intervention

Where the clinician feels there are ongoing safety concerns after the initial assessment, prompt aftercare should be arranged within 48 hours of assessment, with the same professional if possible. This is the time where repeat self-harm is most likely and early follow-up should enhance service engagement, reduce hopelessness, and decrease repeat self-harm and suicide. Continuity of care is valued by clinicians and patients, as well as improving safety.(3) The role of in-patient psychiatric admission was not specifically considered in this guideline. While it can be helpful, even life saving for some patients, views on the relative benefits versus harms vary.

There are significant workforce and resource constraints that affect how the recommendations on aftercare might practically be delivered in real world mental health services. Closer working between liaison psychiatry, psychological therapy services, and primary care teams is critical to develop new models of aftercare, including the consideration of digital delivery.

The Cochrane review on the effectiveness of interventions for self-harm repetition was updated in 2021 and this informed NICE treatment recommendations.(3) There are no pharmacotherapies recommended for self-harm per se, although pharmacological treatment can be used to treat any underlying conditions. The guideline did not consider how treatment for conditions related to self-harm might be prioritised. Where patients have a specific mental illness associated with self-harm, treatment for that condition should be arranged according to clinical judgement and associated guidance. The guideline recommends ‘CBT-informed’ psychological treatments are offered for adults: they should be person-centred and be 4-10 sessions in duration. The scope of ‘CBT informed’ is broad and it encompasses a wide variety of therapeutic approaches including problem solving, interpersonal, and cognitive approaches. In young people who frequently self-harm DBT for adolescents (DBT-A) can be considered. The evidence for DBT-A however was limited by a lack of long-term follow-up data and participant samples.(3)

A challenge for mental health services is having the workforce to deliver these treatments when patients need them. The upskilling of nurses or other practitioners in mental health teams, collaborative working with third-sector agencies, and building on existing provision of psychological therapies in primary care could provide some solutions. The guideline highlights the cost effectiveness of these approaches – they will actually save money in the long term. Psychological treatments should not be withheld from people because of diagnosis, coexisting conditions, or substance misuse.

The development of safety plans should be considered for people who self-harm. The fact that the NICE recommendation was ‘consider’ rather than a stronger recommendation reflected the lack of high quality RCT based evidence; however they are in widespread use, and recent reviews have highlighted potential benefits. Again the ‘how’ is at least as important as the ‘what’. Safety plans should be created collaboratively between people who self-harm and mental health professionals, with input from family members or carers where appropriate. They should be accessible to the person, mental health professionals and others who may be named as a source of support such as the GP, and regularly reviewed. They are dynamic documents which benefit from ongoing updates and should be adapted as needs and circumstances change. There is a role for mental health professionals beyond psychiatric practice: the guidance recommends professionals should support schools in developing support plans for students after self-harm.

Harm minimisation approaches designed to avoid, delay, and reduce future self-harm episodes or complications, are extremely contentious. The guideline steers clear of recommendations on ‘safer self-harm’ and suggests that professionals should always instil hope that a person can move away from self-harm as a coping strategy. The guideline does acknowledge the lack of evidence in this area. However, distraction techniques, wound hygiene, or simple education advice such as on the influence of alcohol and illicit substances on self-harm, may be appropriate in the overall approach to the person’s ongoing recovery, after being agreed with the person and family.

## Research recommendations

The guideline recommends five areas that would benefit from further research to identify effective interventions. These include models of care for young people; approaches to assessment in non-specialist settings; admission to hospital after self-harm; and psychological interventions and harm minimisation approaches.

## Our business and everyone’s business

All mental health professionals will encounter people after self-harm in their daily practice and mental health services remain a key setting for intervention and prevention. However, the updated NICE self-harm guideline reminds us that self-harm can present anywhere across the health and social care system: in education, in criminal justice, and in community settings. This is a public mental health concern that not only impacts mental health services but also wider society.

These recommendations emphasise the importance of compassionate person-centred care which all patients deserve. They highlight the need for a psychosocial assessment for each self-harm episode, the lack of usefulness of risk tools and scales, and the importance of considering the individual needs and safety of the patient, and how safety can be optimised.

Implementation of the guideline is likely to require training across services (perhaps based on existing or updated competency frameworks), protected and regular supervision, and new models of care. These models need to be appropriately tested and quality improvement (QI) approaches may be helpful. The recent ED-SAFE 2 cluster RCT published since the NICE guideline, suggested QI with an emphasis on safety planning led to almost a halving in acute presentations for suicidal behaviour.(5)

Adequate resources, preferably ring-fenced funding, will be required for services to develop new ways of managing self-harm, and in all such developments, the involvement of people with lived experience and the third sector is crucial. In the context of a ‘permacrisis’ where we are emerging from a pandemic into an ongoing economic storm and pressured health systems, implementation will be challenging to say the least. Acting on, and promoting, the new NICE guidance for self-harm will improve care for patients who do not always get the standard of treatment they deserve. But the question is, will we realise this opportunity, or will it be lost?
